# Vestibular Thresholds: A Review of Advances and Challenges in Clinical Applications

**DOI:** 10.3389/fneur.2021.643634

**Published:** 2021-02-19

**Authors:** Megan J. Kobel, Andrew R. Wagner, Daniel M. Merfeld, Jameson K. Mattingly

**Affiliations:** ^1^Department of Otolaryngology – Head and Neck Surgery, The Ohio State University Wexner Medical Center, Columbus, OH, United States; ^2^Department of Speech and Hearing Science, The Ohio State University, Columbus, OH, United States; ^3^Department of Health and Rehabilitation Sciences, The Ohio State University, Columbus, OH, United States

**Keywords:** vestibular system, self-motion perception, vestibular thresholds, psychophysics, vestibular disorders

## Abstract

Vestibular disorders pose a substantial burden on the healthcare system due to a high prevalence and the severity of symptoms. Currently, a large portion of patients experiencing vestibular symptoms receive an ambiguous diagnosis or one that is based solely on history, unconfirmed by any objective measures. As patients primarily experience perceptual symptoms (e.g., dizziness), recent studies have investigated the use of vestibular perceptual thresholds, a quantitative measure of vestibular perception, in clinical populations. This review provides an overview of vestibular perceptual thresholds and the current literature assessing use in clinical populations as a potential diagnostic tool. Patients with peripheral and central vestibular pathologies, including bilateral vestibulopathy and vestibular migraine, show characteristic changes in vestibular thresholds. Vestibular perceptual thresholds have also been found to detect subtle, sub-clinical declines in vestibular function in asymptomatic older adults, suggesting a potential use of vestibular thresholds to augment or complement existing diagnostic methods in multiple populations. Vestibular thresholds are a reliable, sensitive, and specific assay of vestibular precision, however, continued research is needed to better understand the possible applications and limitations, especially with regard to the diagnosis of vestibular disorders.

## Introduction

The vestibular system senses head motion, including rotation, translation and orientation relative to gravity, via input from the semicircular canals (SCC), otolith organs, and their subsequent central integration. Signals from the vestibular periphery have a wide range of reflexive functions, including gaze stabilization via the vestibulo-ocular reflex (VOR), postural control, and autonomic regulation. The vestibular system also contributes to percepts of head motion and spatial orientation, along with contributions from vision, somatosensation, and proprioception. When an injury occurs to the peripheral end organs or central vestibular structures, patients may report abnormal perception of self-motion, imbalance, blurring of vision, and oscillopsia.

Diagnosis and management of patients with vestibular disorders can be challenging due to poor understanding of the underlying pathology, and the lack of reliable objective tests capable of fully evaluating peripheral and/or central vestibular function. Standard physiological assessment of the vestibular system focuses on reflexes including the VOR (i.e., caloric testing, rotary chair, head impulse testing) as an assay of SCC function and vestibulospinal reflexes (VSR) (i.e., vestibular evoked myogenic potentials – VEMPs) as an assay of otolith function. In general, these measures can be effective at localizing lesions or supporting/refuting certain pathologies; however, the results of such tests are often nonspecific to common vestibular pathologies (e.g., vestibular migraine) (1), have poor correlation to patient reported symptoms or perceived disability (2–5), cannot assess the central integration of canal and otolith inputs (6, 7), and have limited physiological relevance (e.g., VEMPs, caloric testing). Furthermore, approximately one-third of patients will have normal or non-localizing results with these tests, suggesting that these tests are inadequate for a thorough evaluation of many vestibular disorders (1).

There is evidence that vestibular perception has qualitatively different underlying mechanisms than vestibular reflexes (8–10), thus serving as a potential source of novel or additive information for those affected by vestibular disorders. This may be particularly important for central disorders (e.g., vestibular migraine), as perceptual tasks have been shown to reflect a higher level of central processing that is otherwise neglected in reflexive assessments (8, 9, 11). Compared with clinical testing of the VOR, which has been widely studied and implemented, much less is known about vestibular perceptual thresholds. Vestibular perceptual thresholds provide a quantitative measure of the smallest self-motion stimulus that can be reliably perceived by an observer (this somewhat terse definition will be expanded upon further below). Although vestibular (i.e., self-motion) perception has been studied for decades, original studies have focused more on studying the non-dynamical aspects of these responses in healthy, rather than symptomatic, populations (e.g., pilots, astronauts) (12–15).

**Figure 1:**
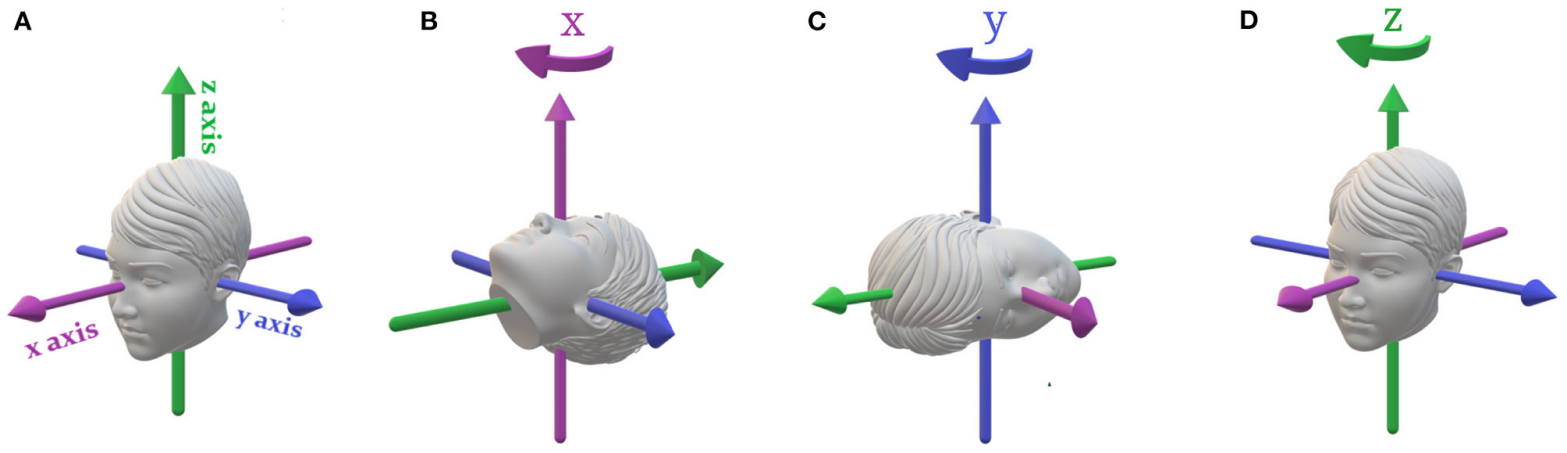
Primary rotations of the head. **(A)** Movement is described in a head-fixed coordinate system. The x-axis is naso-occipital, y-axis is inter-aural, and the z-axis is head-vertical. **(B)** Roll rotation about the x-axis stimulating the vertical SCCs. **(C)** Pitch rotation about the y-axis stimulating primarily the vertical SCCs. **(D)** Yaw rotation about the z-axis stimulating the horizontal SCCs.

**Figure 2:**
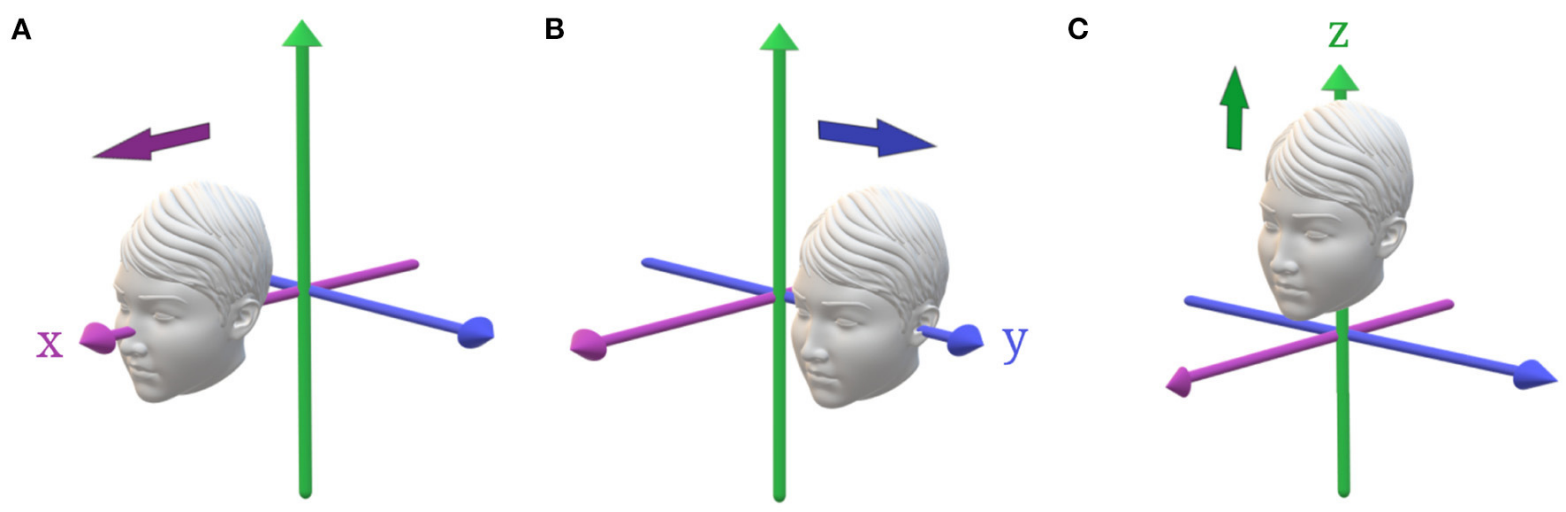
Primary head translations. **(A)** Positive x-translation, along the naso-occipital axis, stimulating predominantly the utricle with saccular contributions. **(B)** Positive y-translation, along the inter-aural axis, stimulating the utricles. **(C)** Positive z-translation, along the head-vertical axis, stimulating the saccules.

**Figure 3:**
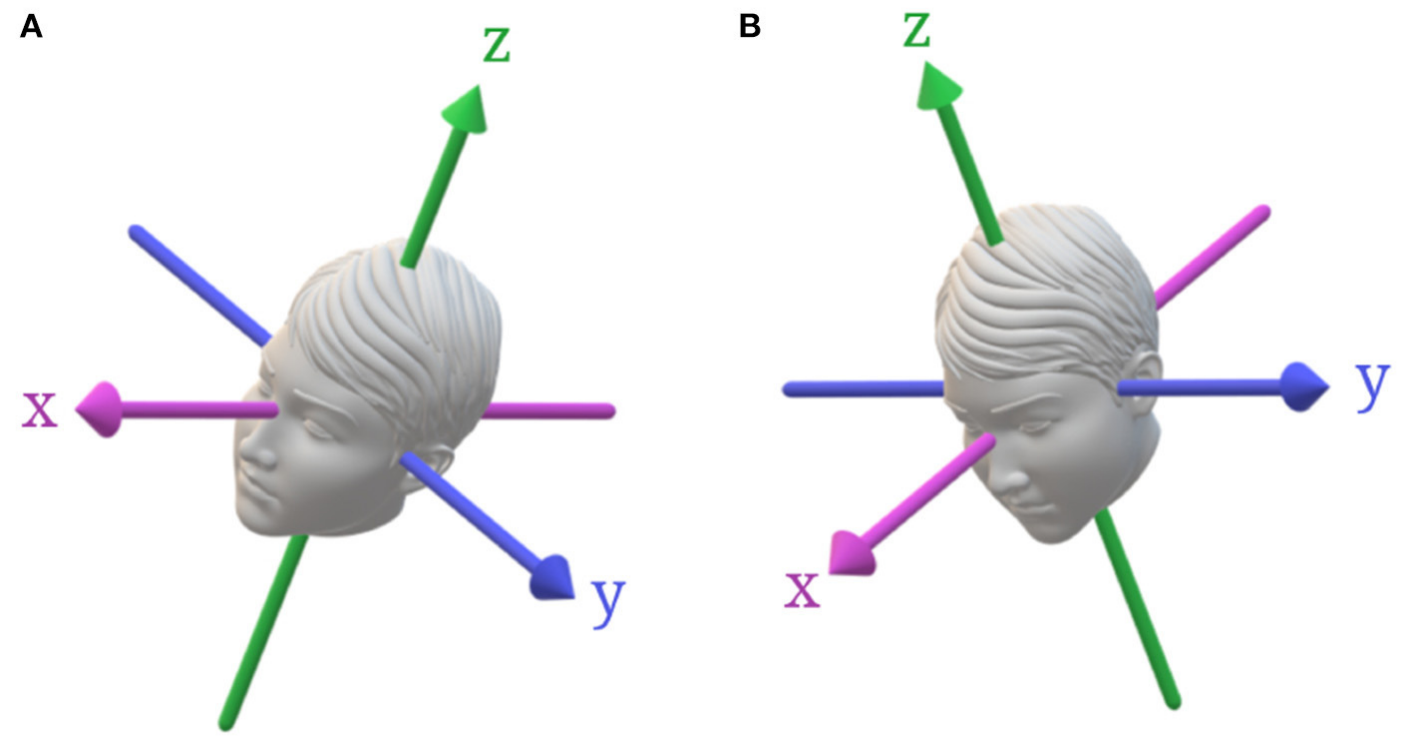
Head tilts from upright with respect to gravity. **(A)** Roll tilt about an earth-vertical axis, which stimulates both the vertical SCCs and the utricle. **(B)** Pitch-tilt about an earth-vertical axis, which stimulates both the vertical SCCs and the otoliths.

Vestibular thresholds can also describe each of the peripheral vestibular organs using a single methodology, both independently – yaw rotation for the horizontal SCC, roll or pitch rotations about an earth vertical axis for the vertical canals (Figure 1), z-axis translation for the saccule, y-axis translation for the utricle, x-axis translations for both the utricle and saccule (Figure 2)—and when SCC and otolith cues interact during rotations (i.e., tilts) about an earth horizontal axis (Figure 3) (6–9). This is a significant advantage over conventional vestibular tests that require multiple devices to thoroughly evaluate the vestibular system - e.g., calorics and rotary chair to evaluate the horizontal SCC and VEMPs to evaluate otolith mediated reflexes (16, 17). Furthermore, from a practical standpoint, testing of the VOR can be quite bothersome to patients, as it can be associated with motion sickness including severe nausea and vomiting, which often limits the ability or willingness to complete testing (18, 19).

While the theoretical benefits of studying vestibular perceptual thresholds is clear, their specific utility with respect to clinical medicine has only recently been examined. Thus, our goal with this review article is to highlight the methodology and clinical contributions that have been published and discuss how these findings may be useful to clinicians in the future, particularly as it pertains to the diagnosis of vestibular disorders.

## Overview of Vestibular Perceptual Thresholds

Vestibular perceptual thresholds refer to the smallest appreciable stimulus, or in this case motion, detected by the participant in some proportion of trials set by the investigator (20, 21). Similarly, in signal detection theory terminology (22), a threshold is the level at which a signal becomes distinguishable relative to noise (21). Studies reviewed herein were limited to those which reported thresholds and not those studying vestibular perception using other supra-threshold stimuli. A brief overview of methods will be provided, but a full review, including application of signal detection theory, adaptive methods, and fitting psychometric functions, is outside the scope of this review and interested readers are directed to Merfeld (21), Lim and Merfeld (23), Chaudhuri and Merfeld (24), and Karmali et al. (25). When measuring thresholds, a recognition task (i.e., left vs. right) is used more commonly than a detection task (i.e., no motion vs. motion) due to the influence of vibration and other cues on detection tasks (21, 26–28). Techniques are common to other psychophysical tasks used to assess other sensory domains, with the participant being provided with a large number of trials traversing a wide range of magnitudes; binary responses (e.g., present/absent, left/right) are then fit to a psychometric function to determine an estimate of threshold based off of pre-determined criteria (e.g., 79.4% correct). To determine magnitude of the test stimuli, a variety of non-adaptive (i.e., predetermined levels) and adaptive (i.e., stimulus changes based on participant responses) have been used. An adaptive staircase, where the subject is required to answer correctly on a predefined number of consecutive trials in order to reduce the stimulus level, is a common paradigm in self-motion perception tasks due to the capacity to accurately and efficiently estimate thresholds (25, 29).

An important aspect to consider is that motion detection is inherently dependent upon multisensory cues, with many extra-vestibular senses contributing to self-motion perception, including vision, somatosensation, proprioception, and audition (6, 30, 31). While whole-body motion thresholds are referred to as “vestibular” thresholds, other modalities have an impact on perceptual thresholds as evidenced by the fact that patients with complete vestibular surgical ablation are able to complete threshold tasks, albeit at thresholds significantly higher (~1.3–56 times) than those without vestibular pathology (32). This obviously complicates evaluation of vestibular perception, and most studies go to great lengths to prevent contributions by non-vestibular cues – including testing in complete darkness, using noise-canceling headphones or active noise cancellation, and taking precautions to minimize localizing tactile feedback (e.g., skin coverage, padding). Methodologic considerations such as the choice of performing recognition, rather than detection, tasks have been emphasized to minimize extra-vestibular vibratory cues (21, 33).

The units of measure used to report vestibular perceptual thresholds has varied between studies; this choice is largely dependent on the test stimuli employed and the targeted end-organ. Many studies report vestibular perceptual thresholds in terms of peak velocity of the motion stimulus [e.g., (27, 34, 35)]; this is common in studies of the SCCs, since the SCC's act as integrating angular accelerometers and the afferent canal signal is proportional to angular velocity (10, 27, 32, 36). Similarly, thresholds for motions stimulating the otoliths are often reported in terms of peak acceleration of the test stimulus [e.g., (20, 26, 37)] since the otolith afferent signal is proportional to net gravitoinertial acceleration [e.g., (38, 39)]. Yet, peak velocity has been reported for translation thresholds [e.g., (32, 35, 40)] and peak acceleration has been reported for rotational stimuli meant to assess canal function [e.g., (41, 42)]. Although standardized units will be beneficial for clinical implementation, many experimental paradigms use stimuli [e.g., single cycles of sinusoidal acceleration; (27, 43)] that allow simple mathematical conversion between reported units permitting direct comparison between studies or between clinics.

## Vestibular Perceptual Thresholds as a Measure of Vestibular Function

### Evaluation of SCC Function

Yaw rotation about an earth-vertical axis has been the most widely studied motion trajectory and primarily reflects horizontal SCC function (Figure 1). Yaw perceptual thresholds reported in the literature have demonstrated significant variability, and this is thought to be related to the type of psychophysical procedure, frequency of the motion stimuli, and differences in equipment used to generate the motion stimulus (26, 27, 44–46). Recently, however, test-retest reliability has been shown to be excellent (intraclass correlation = 0.92) suggesting minimal within subject variation (46). Vertical SCC function is more difficult to measure as this involves applying a roll rotation while avoiding concurrent otolith stimulation during tilts relative to gravity (e.g., roll rotation with the subject in supine, pitch rotations with the subject in ear down; Figure 1) (47). Additionally, vertical canals can be assessed using rotations about an earth vertical axis in the plane of the vertical canals [right-anterior left posterior (RALP) or left-anterior right-posterior (LARP)]; however, this methodology has not been routinely implemented.

Yaw rotation velocity thresholds have been found to display high-pass characters with a characteristic increase below 0.2 Hz and a plateau between 0.5 and 5.0 Hz (7, 27, 40). Benson et al. revealed similar results for yaw rotation, in which thresholds decreased with frequency, but their testing was limited to lower frequency motions (<1.11 Hz) limiting assessment of a high frequency plateau (48). With the frequency range extended to capture the high-pass characteristics, the average cutoff frequency was 0.23–0.44 Hz corresponding to time constants of 0.3–0.15 s, which is significantly shorter than even that of the peripheral vestibular afferents (27, 40). This time constant reduction has been referred to as “velocity leakage,” which is in contrast with the behavioral time constant increase which is commonly referred to as “velocity storage” (27, 32). This velocity plateau suggests that the brain performs the recognition task using velocity rather than position or acceleration information, which is consistent with the assertion that SCC act as integrating angular accelerometers (10, 27, 32, 36). Yaw VOR thresholds, measured using similar techniques as perceptual experiments but measuring eye movements, found that VOR thresholds were not high pass filtered and relatively constant between 0.2 and 5 Hz (10). This de-coupling of the VOR and perception is similarly found in motion paradigms that stimulate the otoliths (8–10) and gives insight into the disparate behavior of the perceptual and motor (i.e., VOR) pathways.

### Evaluation of Otolith Function

The otoliths (saccule and utricle) encode the net gravitoinertial force, the sum of linear acceleration and gravity. Vestibular perceptual thresholds assess otolith function using translations in the naso-occipital or x-axis (predominantly utricle), inter-aural or y-axis (utricle), and superior-inferior or z-axis (saccule) planes (see Figure 2). Additionally, the otoliths can be assessed using quasi-static roll tilt in which the otoliths are stimulated in isolation as the patient is tilted at a velocity below SCC thresholds and the subject is asked to report the direction of the static tilt cue (see Figure 3) (7). Similar to yaw rotation, translation thresholds typically display high-pass characteristics with an increase in thresholds below ~1 Hz (32, 40). Additionally, evidence suggests that saccular afferents are less sensitive than utricular afferents, which has been demonstrated by lower thresholds during interaural compared to superior-inferior translations (20, 33, 35, 49). Evaluation of the saccule also poses unique technical challenges in comparison to the utricle due to issues with ceiling effects of vertical motion and equipment limitations (20, 32).

Vestibular pathology has been shown to exert a greater impact on earth-vertical translations (i.e., parallel to gravity) compared to earth-horizontal translations (i.e., perpendicular to gravity) (32, 35); in contrast, in healthy controls, earth-horizontal and earth-vertical translations have been found to be similar (33). As well, perceptual precision has been shown to be reduced when thresholds are assayed in a non-upright position (e.g., supine or side-lying), atypical of routine human motion (33). Additional research is needed to determine the impact of gravitational cues, body/head orientation, and axis of translation (i.e., inter-aural and superior-inferior) in order to ascertain the factors that impact assays of otolith function in disease and health.

### SCC-Otolith Interactions

One unique aspect of vestibular perceptual testing is the capability to assess the central integration of SCC and otoliths cues. Internal models parse the net gravitoinertial force, encoded by the otoliths, into separate estimates of tilt and translation, using the SCC inputs to estimate head orientation relative to gravity during tilt (8, 50–52). Lim et al. found that dynamic roll tilt thresholds, which require canal-otolith integration, measured at 0.2–0.5 Hz were significantly lower than (1) thresholds measuring SCC (via supine roll) or otolith (via quasi-static roll tilt) precision in isolation and (2) a maximum likelihood estimate; this finding was interpreted as evidence that the perception of dynamic roll tilt stimuli requires both direct sensory inputs and indirect information obtained from the dynamic interaction between the canals and otoliths (7). As will be discussed more below, evidence of an abnormal central integration of canal and otolith cues, as indicated by isolated changes in dynamic roll tilt thresholds, may be useful in the diagnosis of certain central vestibular disorders, particularly vestibular migraine (53, 54).

## Use of Vestibular Thresholds in Patient Populations

### Vestibular Hypofunction

Vestibular hypofunction can result from a broad array of pathologies. Etiologies include medication side effects, post-surgical, neoplastic, autoimmune, Meniere's disease and idiopathic hypofunction. Bilateral vestibular hypofunction (BVH) causes progressive symptoms of imbalance, and in severe cases, oscillopsia; BVH is of particular interest in clinical medicine as it remains a poorly defined chronic disorder, with an unknown etiology (55). The current literature concerning perceptual testing in patients with vestibular hypofunction describe the performance of patients encompassing a range of disease severity, including partial and complete bilateral loss (i.e., post-surgical ablation) (11, 12, 20, 32, 40, 40, 56–59) (see Table 1). Early perceptual assessments in patients with labyrinthine dysfunction used a parallel swing and showed a 10-fold increase in linear motion thresholds in a group of hard of hearing children deemed to have bilateral SCC dysfunction (12). These results have been supported by other studies in both unilateral and bilateral vestibular hypofunction, although the methodology and patient populations have differed dramatically (11, 20, 32, 40, 58, 59, 61, 62). Valko et al. performed the only study to date in patients with complete bilateral vestibular loss (i.e., neurofibromatosis type 2 with bilateral surgical ablation to treat vestibular schwannomas) and assessed motion paradigms assaying multiple end-organs across a wide frequency range (0.5–5 Hz) (32). Overall, the results confirmed that vestibular cues were dominant for self-motion tasks, as thresholds for yaw rotation, superior-inferior (z-axis) translation, inter-aural (y-axis) translation, and head-centered roll tilt about a naso-occipital axis were significantly higher (1.3–56.8 times) in patients than in healthy controls. Threshold changes were smallest for motions with more prominent non-vestibular cues (i.e., roll tilt and inter-aural translation) and greatest for superior-inferior (z-axis) translation, suggesting an impaired ability to differentiate transient self-motion cues from constant gravitational acceleration (32).

**Table 1:** Summary of studies investigating the impact of vestibular hypofunction on perceptual thresholds.

**Study**	**Subjects**	**Stimuli**	**Findings**
Valko et al. (32)	• 3 complete bilateral loss (aged 24–58) • 14 healthy controls (mean age 36, SD: 10)	Single cycle of sinusoidal acceleration	• Yaw rotations (1, 2, and 5 Hz), z-translations (0.3, 0.5, 1, 2, and 5 Hz), y-translations (1, 2, and 5 Hz), and roll tilt (0.05, 0.1, 0.2, 0.5, 1, 2, 5 Hz) thresholds were significantly higher in vestibular loss patients. • Yaw rotations at 0.2 and 0.5 Hz and y-translations at 0.3 and 0.5 Hz could not be completed by loss patients at the highest level generated by the motion platform.
Priesol et al. (40)	• 4 bilateral weakness (reduced calorics, reduced time constant) • 14 healthy controls (mean age 36, SD: 10)	Single cycle of sinusoidal acceleration	• Yaw rotation thresholds (0.2, 0.5, 1, 2, and 5 Hz) were significantly higher in bilateral hypofunction; y-translation: (0.3, 0.5, 1, 2, and 5 Hz) were statistically higher, however, the effect was limited to unspecified “lower frequencies.” • Z-translation (0.3, 0.5, 1, 2, and 5 Hz) and roll tilt (0.05, 0.1, 0.2, 0.5, 1, 2, 5 Hz) thresholds were not significantly different between groups.
Shayman et al. (60)	• 3 bilateral weakness (reduced calorics, 35–55 years) • 13 healthy controls (23–49 years)	Single cycles of raised cosine velocity	• Yaw rotation (1 Hz) thresholds were significantly higher in patiens with bilateral weakness.
Agrawal et al. (20)	• 33 bilateral weakness (reduced calorics or HIT, 24–83 years) • 42 healthy controls (15–72 years)	Raised cosine velocity profile	• Z-translation (0.5 Hz), y-translation (0.5 Hz), x-translation (0.5 Hz) thresholds were significantly higher in patients with bilateral vestibular loss.
Bringoux et al. (61)	• 4 bilateral vestibular loss (37–60 years) • 12 healthy controls (mean age: 29 ± 6 years)	Tilts from upright at 0.05 deg/s	• Roll and pitch tilt thresholds were not significantly different between groups.
Gianna et al. (56)	• 5 bilateral vestibular loss (31–64 years) • 8 health controls (24–49 years)	Acceleration steps	• Y-translation thresholds were not significantly different between groups.
Cousins et al. (58)	• 25 VN patients, (mean age: 46) • 30 healthy controls (mean age: 42)	Acceleration at 0.5 deg/s/s, increasing 0.5 deg/s/s every 3 s	• Ipsilesional and contralesional yaw rotation thresholds were significantly higher in VN patients at acute (1–5 days) and recovered (6–16 weeks) time points.
Cutfield et al. (59)	• 12 patients with VN (mean age: 50.0) • 12 healthy controls (mean age: 46.0)	Acceleration at 0.5 deg/s/s, increasing 0.5 deg/s/s every 3 s	• Ipsilesional and contralesional yaw rotation thresholds were significantly higher in VN patients.

*HIT, head impulse test; VN, vestibular neuritis*.

While this is the only study to date to include patients with complete vestibular loss, several studies have assessed vestibular perceptual thresholds in patients with incomplete bilateral vestibulopathy and have identified deficits in perception consistent with varying degrees of end organ dysfunction (11, 20, 40). Yaw rotation thresholds were found to be significantly increased in patients with idiopathic or ototoxic bilateral horizontal SCC dysfunction, as identified by a decrease in gain on caloric testing (40, 60). Priesol et al. (40) also found a modest but statistically significant elevation in inter-aural translation (y-translation) thresholds, possibly reflecting the shared innervation of the horizontal SCC and utricle; however, no significant differences were noted in superior-inferior translations or dynamic roll tilt thresholds. Importantly, these data suggest that end organ pathology in conditions such as idiopathic bilateral hypofunction is non-uniform, and conventional testing in many scenarios may incompletely characterize end-organ pathology. However, generalizability of these results may be limited due to the small sample size and the absence of a comparison test of utricular dysfunction (i.e., VEMPs) (40).

Translation thresholds were also assessed by Agrawal et al. in a group of patients with bilateral horizontal SCC weakness identified via calorics and/or head impulse testing (20). Thresholds for 0.5 Hz naso-occipital (x-axis), interaural (y-axis), and superior-inferior (z-axis) translations were significantly higher in patients than in healthy controls. An association was also noted between vibration-evoked ocular vestibular evoked myogenic potentials (oVEMP) and 0.5 Hz naso-occipital and inter-aural thresholds, suggesting that both tests assay underlying utricular function. Significant associations were not seen between cVEMP findings with any translation threshold, suggesting a dissociation between measures of presumed saccular function (20). However, Bremova et al. noted an opposite pattern in patients with Meniere's disease, showing an association between cVEMPs and 1 Hz superior-inferior (z-axis) and naso-occipital thresholds (x-axis), but no relationship between oVEMPs and any linear translation thresholds (37). The lack of agreement between these studies may reflect differences in the frequency of the test stimulus (0.5 vs. 1 Hz) or differences in the study populations; however, more testing is needed in these areas to further understand these relationships in both healthy and patient populations. It should be noted that other studies have refuted these findings, finding no or minimal difference between labyrinthine defective individuals and normal controls (56, 61). All of these studies have however demonstrated significant methodological heterogeneity, including different motion stimuli, testing frequencies, etiologies and severities of labyrinthine dysfunction.

Notably, determining laterality of vestibular responses in those with a vestibular injury is obscured by the fact that motion stimuli stimulate both labyrinths simultaneously, thus limiting application of published methodologies when lateralization of pathology is needed. Vestibular detection thresholds using yaw acceleration steps revealed asymmetrically elevated thresholds for ipsi-lesionally directed stimuli when testing in the acute stage (1–5 days post onset of vestibular neuritis); however, these thresholds become symmetric within weeks of onset, despite lack of recovery of calorics, revealing a persistent asymmetry in peripheral function (58). As well, while ipsi-lesional rotations may reveal acute changes in perception, this may reflect the central processing of both ipsi- and contra-lesional vestibular systems rather than a signal from the damaged labyrinth in isolation (58, 59).

### Episodic Vestibular Disorders

Several studies have investigated changes in vestibular thresholds seen in episodic vestibular disorders, namely vestibular migraine (VM) and Meniere's Disease (MD) (see Table 2). Vestibular migraine (VM) is estimated to be the most common cause of recurrent episodic vertigo (64, 65), with a prevalence between 1 and 2.7% of the adult population (66). VM is characterized by recurrent episodes of vestibular symptoms in association with signs and symptoms of migraine, including headache, visual aura, photophobia, and phonophobia (67). Due to the frequent reports of positional and head-motion induced symptoms in VM and the characteristic hypersensitivity to sensory stimuli in migraine, possible abnormalities in vestibular sensory perception have been investigated as a putative biomarker (23, 37, 53, 54, 63, 68). Overall, increases in vestibular sensitivity and abnormalities across motion profiles are inconsistent (37, 53, 54, 63, 68); increased sensitivity to motions stimulating both the SCC and otoliths have instead been consistently reported (53, 54, 63, 68).

**Table 2:** Summary of studies investigating the impact of episodic vertigo on perceptual thresholds.

**Study**	**Subjects**	**Stimuli**	**Findings**
Bremova et al. (37)	• 27 Meniere's disease (mean age: 58) • 20 vestibular migraine (mean age: 40.9) • 34 healthy controls (mean age: 44.6)	Raised cosine velocity profile	• Z-translation (1 Hz), and x-translation (1 Hz) thresholds were significantly higher in MD in comparison to both VM and healthy controls. • Y-translation (1 Hz) thresholds were significantly higher in MD in comparison to VM, but not significantly different in comparison to healthy controls.
King et al. (63)	• 12 vestibular migraine (35.5 ± 2.7 years) • 12 migraine (34.0 ± 3.1 years) • 12 healthy control (38.1 ± 3.1 years) • 8 Meniere's disease	Single cycle of sinusoidal acceleration	• Roll tilt thresholds were significantly lower in VM patients in comparison to healthy controls, migraine (0.03, 0.05, 0.1 Hz) and MD (0.2 Hz); no differences were seen at higher frequencies (0.2, 0.5, 1, 2, 5 Hz). • Y-translation (0.2, 0.3, and 0.5 Hz) and roll rotation (0.2 and 0.5 Hz) thresholds not significantly different between VM, migraine, and healthy controls.
Lewis et al. (53, 54)	• 8 vestibular migraine (35.5 ± 2.7 years) • 8 migraine (34.0 ± 3.1 years) • 8 healthy control (38.1 ± 3.1 years)	Single cycle of sinusoidal acceleration	• Roll tilt thresholds (0.1 Hz) were significantly lower in VM in comparison to migraine and healthy controls. • Quasi-static roll tilt (constant ramp of 0.125 deg/s) and roll-rotation thresholds (0.1 and 1 Hz) were not significantly different between patients with VM and healthy controls.
Bednarcazuk et al. (42)	• 15 vestibular migraine (mean age, 42.0) • 15 migraine (mean age: 38.7) • 15 BPPV (mean age: 44.7) • 15 healthy controls (mean age: 44.7)	Acceleration at 0.3 deg/s/s, increasing by 0.3 deg/s/s every 3 s	• Yaw rotation thresholds were significantly higher in VM and BPPV in comparison to patients with migraine and healthy controls.

*BPPV, benign paroxysmal positional vertigo; HIT, head impulse test; MD, Meniere's Disease; VM, vestibular migraine; VN, vestibular neuritis*.

Translation thresholds for naso-occipital (x-axis), inter-aural (y-axis) and superior-inferior (z-axis) motions were not significantly different between patients with VM and healthy controls (37). Consistent with this finding, thresholds were similar between healthy controls, migraineurs without vestibular symptoms, and VM subjects for supine roll rotation (vertical SCCs) and a “quasi-static” roll tilt (otoliths) (53, 54, 63). However, in an experiment using six trials of progressively accelerating rotational stimuli, Bednarczuk et al. reported an increased time (i.e., increased temporal threshold) to perceive yaw angular acceleration in patients with VM and in those with non-migrainous vertigo compared to healthy controls and non-vertiginous migraineurs (42).

In an apparently contradictory finding, a significant decrease in roll tilt thresholds has been demonstrated for VM patients in comparison to both healthy and non-vertiginous migraine controls (53, 54, 63). This reduction in thresholds was only seen with low to mid-frequency stimuli, reflecting an increased sensitivity to combined activation of SCC and otolith cues, given normal thresholds at higher frequencies, where the response reflects predominantly SCC cues (6, 7, 53, 54, 63). King et al. (63) also identified two populations of VM patients with low roll tilt thresholds, with one subset showing a positive correlation between tilt threshold and symptom severity, and the other with thresholds being independent of symptoms. Lower roll tilt thresholds were also shown to correlate with a decrease in VOR time constant in a subset of patients, suggesting sensitization of the cerebellar nodulus and uvula, the presumed site of SCC and otolith integration (63, 69). Abnormal central integration of otolith and SCC cues in VM patients was also found using a centrifugation paradigm, where patients with VM were found to have a slowed perception of roll tilt when presented with conflicting SCC and otolith cues (68, 70). Currently, no pathognomonic finding exists for VM, thus the potential use of low to mid-frequency roll tilt vestibular thresholds to assess midline cerebellar structures is a promising avenue for clinical diagnosis and management.

Vestibular thresholds have also been assessed in Meniere's disease (MD), another frequently encountered episodic vestibular disorder. MD is characterized by episodic vertigo and auditory symptoms, which include fluctuating hearing loss, aural fullness, and tinnitus (71). Histopathological studies have shown that MD can cause damage throughout the cochlea and labyrinth, particularly within the saccule (72). Currently, there is a paucity of research assessing perceptual thresholds in MD. At this time, only two studies have assessed vestibular thresholds in patients with MD (37, 63). Bremova et al. (37) found that MD patients displayed elevated translation thresholds for naso-occipital (x-axis) and superior-inferior (z-axis) translations when compared to healthy controls, suggesting saccular damage. In the study by King et al. (63) patients with MD were found to have normal roll tilt thresholds at 0.2 Hz, contrasting the selective reduction in low to mid-frequency roll tilt thresholds in patients with VM. In addition, Bremova et al. (37) found that translation thresholds in all axes were significantly higher in MD than VM patients, with the largest difference for superior-inferior and naso-occipital axes, even after accounting for age as a covariate. Receiver operating characteristic curve (ROC) analyses assessing differentiation of VM and MD revealed fair to good area under the curve (AUC) values (0.775–0.848) for all three axes of translation, suggesting that vestibular thresholds assessing otolith function may allow separation of these two episodic vestibular disorders (37).

## Vestibular Thresholds as a Maker of Age-Related Vestibular Decline

Degradation of vestibular function with age has been well documented in the literature (73–78); such declines occur alongside an age-associated reduction in the number of vestibular hair cells (79, 80) and vestibular afferent neurons (81). However, the impact of age on rotation and translation perceptual thresholds is less clear (summarized in Table 3). Overall, changes in rotation thresholds reflecting SCC function have been less consistently reported than translation thresholds. The largest study to date assessed vestibular perceptual thresholds in 105 adults across a large age range (aged 18–80) (35). The main finding was that thresholds for 0.2 Hz roll tilt and 1 Hz inter-aural translation (y-axis), superior-inferior (z-axis) translation, roll tilt, and yaw rotation were stable below the age of 42 but showed a significant, monotonic increase above 42 years of age. While all thresholds increased, the largest increase was seen in z-translation thresholds, which increased ~83% above baseline per decade, followed by 1 Hz roll tilt (increase of 56% per decade), y-translation (increase of 46% per decade), 0.2 Hz roll tilt (increase of 32% per decade), and yaw rotation (increase of 15% per decade) (35). Principal component analysis of this dataset revealed that ~20% of the variation in the population was explained by aging and 40% by a single component that included similar contributions from all thresholds (84). This single component was suggested by the authors to represent higher or lower thresholds as an individual trait that may represent physiologic age or anatomic variation across the population (84). It should also be noted that upon re-analysis in which fits were made for each motion trajectory, yaw thresholds no longer demonstrated a statistically significant age effect (84).

**Table 3:** Summary of studies investigating the impact of aging on perceptual thresholds.

**Study**	**Subjects**	**Stimuli**	**Findings**
Seemungal et al. (41)	• 14 young (19–37 years) • 9 older (56–75 years)	Triangular velocity profile, 10 s	• Yaw rotation thresholds were not significantly different between young and older adults.
Chang et al. (82)	• 19 young (20–26 years) • 16 older (63–84 years)	5 s of sinusoidal rotations	• Yaw rotation thresholds were not significantly different between young and older adults.
Kingma (83)	• 28 subjects (22–60; seven/decade)	Raised sinusoids (5 periods maximum)	• X-translation thresholds (1 Hz) showed a significant increase with age. • Y-translation thresholds (1 Hz) did not show a significant increase with age.
Roditi and Crane (34)	• 16 younger adults (21–49) • 8 older adults (50–8 years)	Single cycle of sinusoidal acceleration	• Z-translation (0.5 and 1 Hz), y-translation (0.5 and 1 Hz) and x-translation (0.5 Hz) thresholds were significantly higher in older adults compared to younger adults. • Yaw rotation (0.5 and 1 Hz) and x-translation (1 Hz) thresholds were not significantly different between younger and older adults.
Agrawal et al. (20)	• 42 healthy controls (15–72 years)	Raised cosine velocity profile	• Z-translation (0.5 Hz), y-translation (0.5 Hz) and x-translation (0.5 Hz) thresholds showed a significant positive correlation with age.
Bremova et al. (37)	• 34 healthy controls (mean: 44.6 years, SD: 15.2)	Raised cosine velocity profile	• Z-translation (1 Hz), y-translation (1 Hz) and x-translation (1 Hz) thresholds showed a significant positive correlation with age.
Bermudez et al. (37), Karmali et al. (84), and Beylergil et al. (85)^a^	• 105 subjects (18–80 years)	Single cycle of sinusoidal acceleration	• Z-translation (1 Hz), y-translation (1 Hz), and roll tilt (0.2 and 1 Hz) thresholds were constant below ~42 years of age and displayed a significant monotonic increase between 42 and 80. • Yaw rotation thresholds (1 Hz) did not show significant increases with age when examined in isolation by Karmali et al. (84).

a *The dataset in Bermudez et al. (35) was subsequently further examined by Karmali et al. (84) and Beylergil et al. (85), thus studies are summarized together*.

Similarly, several other studies have failed to detect a significant increase in yaw rotation thresholds with age. Seemungal et al. found similar yaw acceleration thresholds between healthy young adults (aged 19–37) and older adults (aged 56–75) using a triangular velocity trajectory (86). Likewise, no differences were noted in 0.5 Hz yaw detection and discrimination thresholds between younger (aged 20–26 years) and older (aged 63–84) adults (82), and for 0.5 Hz recognition thresholds in younger (age <50) and older adults (age > 50) (34). These findings suggest that yaw rotation may be impacted differently by aging than other profiles which display clear aging effects. While moderate correlation coefficients have been demonstrated between all five motion profile thresholds, even after adjusting for age, the lowest coefficients were between yaw and any translation or roll tilt threshold (84). This provides additional evidence that yaw earth-vertical rotational cues are processed differently than other motion paradigms. For example, yaw rotations about an earth-vertical axis only receive useful information from the horizontal SCC, while translations and tilt require central integration of SCC and otolith cues to disambiguate tilt from translation cues (8, 9, 84). Additionally, there is evidence that yaw rotation and horizontal SCC stimulation may undergo more extensive or unique central processing due to the longer time constant when compared to the vertical SCCs (87) and the reduced impact of otolith cues on velocity storage (88).

The preferential impact of age on thresholds stimulating the otoliths demonstrated by Bermúdez Rey et al. (35) is also reflected in a number of studies that have detected age-related changes in translation thresholds, specifically for trajectories assaying saccular function (20, 34, 37, 83). In subjects aged 15–83, 0.5 Hz naso-occipital (x-axis) and superior-inferior (z-axis) perceptual thresholds were found to be significantly correlated with age, but inter-aural thresholds did not demonstrate this same relationship (20). Similarly, Kingma (83) reported that in contrast to naso-occipital axis thresholds, 1 Hz inter-aural translation thresholds did not correlate with age. However, Roditi and Crane (34) compared adults below and above the age of 50, and saw a significant difference in 0.5 and 1 Hz inter-aural and superior-inferior thresholds and 0.5 Hz naso-occipital thresholds. While 1 Hz naso-occipital thresholds failed to reach a statistically significant difference between younger and older adults, this may have been reflective of the small sample of older adults in this study (*n* = 3), as another study of 34 healthy subjects saw a significant positive correlation with age for 1 Hz naso-occipital, inter-aural, and head vertical translation thresholds (37).

While studies measuring yaw and translational thresholds have shown mixed findings, assessment of roll tilt thresholds have revealed unique insights into the influence of vestibular function on age-related balance impairment (35, 84, 85). An increase in 0.2 Hz roll tilt thresholds was shown to be accompanied by a significant increase in the risk of balance impairment as assessed by the inability to complete a foam surface eyes closed balance task (35, 84, 85), a finding previously shown to predict more than a six-fold increase in fall risk (35, 78, 84, 85). Subsequent mediation analyses found that 0.2 Hz roll tilt thresholds mediated approximately 46% of the relationship between age and balance impairment (85). While this relationship needs to be further explored, these results suggest a potential future clinical application of roll tilt thresholds as a mechanism to identify age-related balance declines and fall risk.

## Discussion

The study of vestibular perception traverses many scientific domains, spanning from the study of spatial disorientation in pilots to the differential diagnosis of vestibular disorders. This review, however, employs an intentionally narrow focus. The inherent limitations of current vestibular function tests have prompted this review to explore the state of the evidence as it pertains to the use of vestibular perceptual thresholds in clinical medicine.

Vestibular perceptual thresholds have the capacity to quantify the integrity of each vestibular end organ (otoliths and canals), a substantial improvement upon current vestibular assessments. As an example of possible clinical utility, Priesol et al. was able to show a specific pattern of end-organ damage in individuals with idiopathic bilateral vestibular hypofunction, which included elevated thresholds during yaw rotation and low frequency interaural translation (40). Routine clinical testing would have incompletely characterized the specific pattern of end-organ dysfunction in these individuals due to an inability to individually survey the peripheral vestibular apparatus independent of the extra-vestibular factors that influence VOR and VEMP responses. The natural vestibular stimulus, head rotation and/or translation, used by threshold assessment may also explain the finding that thresholds, but not standard vestibular function tests, correlate with patient symptoms (2, 63).

Vestibular thresholds may also be useful to assess treatment response or disease progression for those with unilateral or bilateral vestibular hypofunction (20, 58). Standard metrics, such as VOR gain, are limited in their ability to closely monitor vestibular function due to the compensatory recruitment of oculomotor strategies (89–91). Recently, test re-test reliability for vestibular threshold testing was shown to be very reliable, suggesting a potential to use thresholds to track vestibular function over time (46, 92). Furthermore, the results of traditional vestibular function tests often do not correlate with the extent of one's perceived dizziness related handicap (2). Positive correlations have however been identified between perceptual thresholds and dizziness handicap inventory (DHI) scores in subjects with vestibular migraine and bilateral vestibular hypofunction (20, 63). The independence of thresholds on oculomotor function may also prove particularly useful in measuring vestibular function in those with oculomotor disorders (e.g., congenital nystagmus), as these conditions impact traditional vestibular tests of the VOR (62).

Differentiation between other, more ambiguous vestibular disorders (e.g., MD and VM) appears particularly promising. Many patients with vestibular disorders present with symptoms that result from an unknown etiology, without clear indication of a specific disease process or of an individually culpable vestibular organ. Traditional diagnostic methods are typically exclusionary, excluding the more obvious etiologies prior to confirming a diagnosis based upon patient symptoms (67, 71). However, recent findings suggest that mid to low frequency roll tilt perceptual thresholds may serve as a biomarker for VM, suggesting that vestibular thresholds may provide an objective metric to differentiate VM from other episodic vestibular disorders with similar symptom profiles, namely MD (53, 54, 63). Considering the findings both in VM and in vestibular hypofunction, these results suggest a broadened capacity for thresholds to be used as a critical piece to the diagnostic puzzle in patients with ambiguous symptoms of central or peripheral etiology (53, 54, 63).

The comprehensive nature of vestibular thresholds also allows for potential improvements in our understanding of how age influences vestibular function. This point is not trivial, given the well-documented association between aging, vestibular decline, and fall risk (73–78). Roll tilt thresholds at 0.2 Hz in particular have been shown to predict the likelihood of failing condition four of the modified Romberg balance test (eyes closed, compliant stance balance task), an outcome previously shown to be associated with a 6.3-fold increase in the odds of falling (78) in older adults. As mentioned above, a standard mediation analysis of the same data set found that 0.2 Hz roll tilt thresholds, accounted for nearly half of the well-known association between aging and fall risk (85). Although we agree that this does not imply causation, this finding does suggest that if one were to consider all of the alternative factors likely to contribute to age-related balance dysfunction (e.g., proprioception, cognition, strength), the combined effect of these factors would be approximately equal to the contribution of a single variable, roll tilt perceptual thresholds. Although these results are in need of confirmation in additional samples of older adults, at a minimum, vestibular noise, assayed by roll tilt perceptual thresholds, appears to be one of the primary contributors to age-related balance decline and fall risk. It is worth noting that the aforementioned findings were made in *asymptomatic* adults without complaints of vestibular impairment, suggesting that roll tilt thresholds may prove to be a sensitive screening tool to detect sub-clinical vestibular impairment and fall risk in asymptomatic adults over age 40.

From a logistical standpoint, an advantage of vestibular perceptual testing is the relative ease of testing, the task is simple and intuitive and can be readily learned by most, if not all, patients. This testing is similar to a standard hearing test, which may be the most commonly performed threshold procedure. Furthermore, algorithms that yield efficient data collection and precise data analysis have already been automated, making it possible for non-specialists to perform testing with minimal training. Unlike the heterogeneity in some other vestibular tests, this automation may serve to help standardize procedures across laboratories.

Several methodological limitations do however influence the potential clinical use of vestibular thresholds. The principal limitation with vestibular perceptual threshold testing is the time and equipment required to perform an accurate assessment. This is particularly an issue with lower frequency testing where each individual motion can require a significant amount of time (e.g., 0.1 Hz takes 10 s for 1 cycle). Automatic computer-based threshold environments and adaptive methodological approaches (e.g., staircase paradigms) have reduced test durations, yet the average assessment still requires between 10 and 20 min (~100 trials) per test motion. We cannot directly observe one's internal perception of a sensory stimulus, and instead we are forced to rely upon a subjective report of their perceived world state (e.g., “I feel that I moved left”). Thus, aside from logistical concerns, the increased time for threshold testing introduces potential errors related to subject inattention and fatigue. This problem can be mitigated by ensuring the subject receives adequate rest, that testing occurs at a time of day where the subject is more alert, and by using statistical techniques that exclude attentional lapses from the threshold analysis (93, 94).

In addition, particular care must be taken to avoid the introduction of potentially confounding extra-vestibular cues [auditory, visual, and somatic (e.g., vibration)]. Veridical visual cues (6) and earth fixed auditory cues (30) have each been shown to reduce perceptual thresholds, and thus can influence vestibular thresholds if visual and auditory cues are not adequately controlled. When using a motion platform to deliver stimuli, somatic cues such as vibration are unavoidable. However, using a direction recognition task (e.g., did I move right or left?) rather than a detection task (e.g., did I move) can mitigate the effect of vibration on vestibular thresholds (21, 26, 28).

From an equipment standpoint, vestibular perceptual threshold measurements require only a few simple components (i.e., a motorized chair, a tablet or subject response buttons, and a computer for device control and data acquisition). Yaw perceptual thresholds could be performed using a rotary chair, which is found in most tertiary care vestibular referral centers and audiology clinics. However, immediate implementation is not feasible with most commercially available systems and will be dependent upon thedevelopment of appropriate software and hardware by the device manufacturers. The primary limitation of the rotary chair in comparison to a 6DOF motion platform is the limitation in test conditions, as the rotary chair can only be used to assess yaw thresholds within a limited frequency range. Therefore, a motion platform with multiple degrees of freedom is likely necessary for a comprehensive assessment of vestibular thresholds. While currently 6DOF motion platforms (e.g., Moog) are not commonly available, we feel that their implementation would be very straightforward. This equipment could fit in a small room and the total cost is estimated to be < $200 K, an estimate that is based upon our own lab set-up. We emphasize that all aspects of both central and peripheral vestibular function would be tested using the single motion device, and as a result this equipment would almost certainly cost less than the total cost of the devices currently used today (rotary chair, caloric irrigator, evoked potentials equipment, head impulse goggles, etc.).

## Summary

Vestibular thresholds are arguably the most direct, sensitive, and specific assay of vestibular noise currently available (20, 63, 95). The ability to test all end-organs and their central integration, the correlation to patient symptoms, the possible role in differentiating certain vestibular disorders, and the relative ease of testing make thresholds a promising clinical measure. Continued research is needed to better understand the possible applications and limitations, especially with regard to the differential diagnosis of vestibular disorders. Such disorders continue to be a challenge to manage clinically and the absence of reliable diagnostic testing is a critical barrier to improving the day-to-day management of these patients.

## Author Contributions

MK, AW, DM, and JM wrote the manuscript and approved the final version prior to publication. MK created all tables and figures presented herein. All authors contributed to the article and approved the submitted version.

## Conflict of Interest

The authors declare that the research was conducted in the absence of any commercial or financial relationships that could be construed as a potential conflict of interest.
